# Crowdsourcing Participatory Evaluation of Medical Pictograms Using Amazon Mechanical Turk

**DOI:** 10.2196/jmir.2513

**Published:** 2013-06-03

**Authors:** Bei Yu, Matt Willis, Peiyuan Sun, Jun Wang

**Affiliations:** ^1^School of Information StudiesSyracuse UniversitySyracuse, NYUnited States

**Keywords:** crowdsourcing, Amazon Mechanical Turk, participatory design, medical instruction, pictogram, patient communication, readability, health literacy

## Abstract

**Background:**

Consumer and patient participation proved to be an effective approach for medical pictogram design, but it can be costly and time-consuming. We proposed and evaluated an inexpensive approach that crowdsourced the pictogram evaluation task to Amazon Mechanical Turk (MTurk) workers, who are usually referred to as the “turkers”.

**Objective:**

To answer two research questions: (1) Is the turkers’ collective effort effective for identifying design problems in medical pictograms? and (2) Do the turkers’ demographic characteristics affect their performance in medical pictogram comprehension?

**Methods:**

We designed a Web-based survey (open-ended tests) to ask 100 US turkers to type in their guesses of the meaning of 20 US pharmacopeial pictograms. Two judges independently coded the turkers’ guesses into four categories: correct, partially correct, wrong, and completely wrong. The comprehensibility of a pictogram was measured by the percentage of correct guesses, with each partially correct guess counted as 0.5 correct. We then conducted a content analysis on the turkers’ interpretations to identify misunderstandings and assess whether the misunderstandings were common. We also conducted a statistical analysis to examine the relationship between turkers’ demographic characteristics and their pictogram comprehension performance.

**Results:**

The survey was completed within 3 days of our posting the task to the MTurk, and the collected data are publicly available in the multimedia appendix for download. The comprehensibility for the 20 tested pictograms ranged from 45% to 98%, with an average of 72.5%. The comprehensibility scores of 10 pictograms were strongly correlated to the scores of the same pictograms reported in another study that used oral response–based open-ended testing with local people. The turkers’ misinterpretations shared common errors that exposed design problems in the pictograms. Participant performance was positively correlated with their educational level.

**Conclusions:**

The results confirmed that crowdsourcing can be used as an effective and inexpensive approach for participatory evaluation of medical pictograms. Through Web-based open-ended testing, the crowd can effectively identify problems in pictogram designs. The results also confirmed that education has a significant effect on the comprehension of medical pictograms. Since low-literate people are underrepresented in the turker population, further investigation is needed to examine to what extent turkers’ misunderstandings overlap with those elicited from low-literate people.

## Introduction

The Department of Health and Human Services defines health literacy as “the degree to which individuals have the capacity to obtain, process, and understand basic health information and services needed to make appropriate health decisions” [1]. This concept of health literacy is prevalent in the written materials a patient may receive at a hospital, in pharmaceutical instructions, verbal instructions, and any health information encountered online.

Lengthy, purely text-based medical instructions have been reported to result in poor patient attention, comprehension, recall, and adherence. This challenge is particularly acute for patients with low literacy levels, since medical instructions are commonly written at a level exceeding the average American’s reading level, and the average reading level is even lower in certain regions, like inner cities and impoverished areas [2,3].

Many interventions have been designed to improve patients’ understanding of medication. One promising approach is to add pictorial aids or pictograms to patient information materials. Many studies have shown that pictograms can enhance text-based instructions by increasing patients’ attention to the instructions and their comprehension and recall of the content details [2-10].

Studies have also shown that for pictograms to effectively communicate medical instructions, consumers, patients, and health professionals should be involved in the process of iterative design and testing [10-12]. However, the cost for participatory design can be high, considering the variety of medical instructions and the time expenditure for patients, health professionals, and designers. Therefore, to date, participatory design studies have been conducted only on a small scale [2,7,9].

Crowdsourcing, with its low cost of recruiting participants and almost immediate access to a large number of Internet users, provides an attractive option for participatory design and evaluation of medical pictograms [13-18]. We envisioned building a crowdsourcing tool in which Internet users could create a variety of pictograms for any medical instruction, and then the best pictograms would be selected by the crowd and be evaluated for their comprehensibility.

Our concept has two critical components: crowdsourced pictogram design and crowdsourced evaluation. In this study, we focused on the crowdsourced evaluation. Specifically, we aimed to assess the comprehensibility of standard US Pharmacopeial Convention pictograms using Amazon Mechanical Turk (MTurk). With hundreds of thousands of turkers from over 100 countries, MTurk can help recruit a large number of diversified turkers to work on microtasks in a very short time period at a very low cost, such as a few cents per user response.

Our research questions are: (1) Is the turkers’ collective effort effective for identifying design problems in medical pictograms? and (2) Do the turkers’ demographic attributes affect their performance in medical pictogram comprehension? We hypothesized that turkers would be able to identify common design problems in medical pictograms. We also expected that turkers with higher educational level and caregivers would perform better in this task.

## Methods

Our study consisted of three steps: first, searching for samples of medical pictograms; second, programming and deploying the Web-based survey; and third, setting up our survey on MTurk. This section describes the details of each step of the survey set-up.

### Selecting the Medical Pictograms

**Figure 1:**
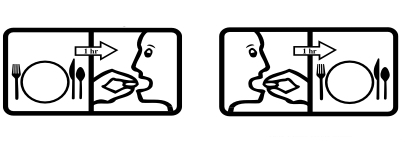
Pictogram redundancy.

**Figure 2:**
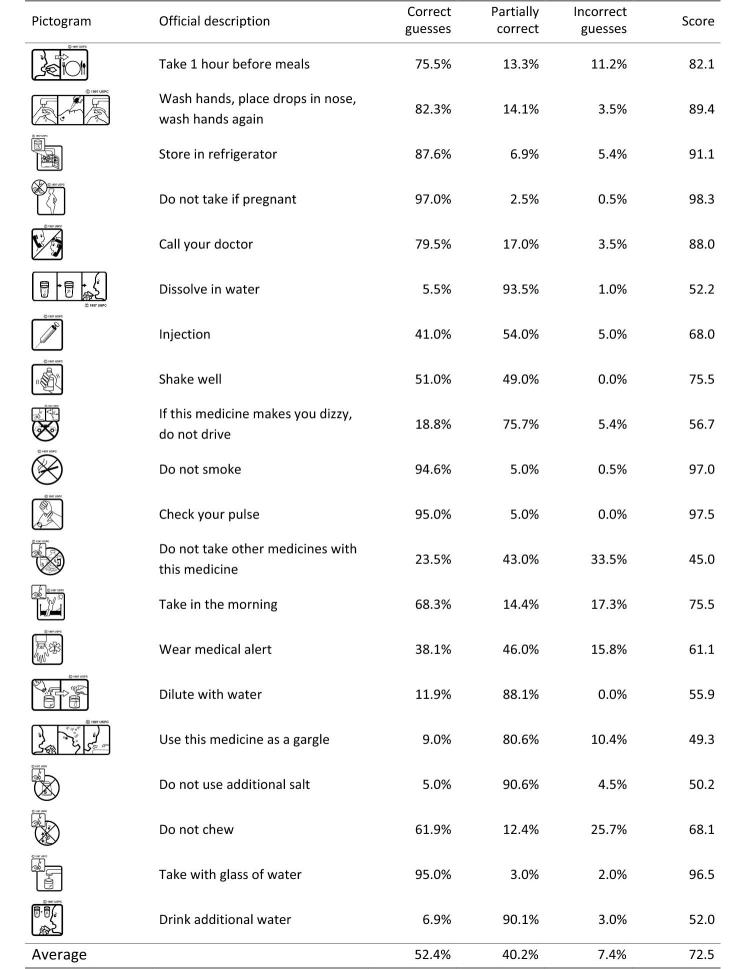
The 20 pictograms and their comprehensibility scores.

The ideal pictogram candidates for this evaluation study would be pictograms that were standardized, freely available for others to use, and found on US pharmacological products. Based on these criteria, we chose to use the US Pharmacopeial Convention’s Pictogram Library as the set of pictograms for evaluation. The pictogram library contains 81 pictograms and can be downloaded for free from their website. Many of the pictograms include identical or similar elements. For example, the only difference in the two pictograms in Figure 1 is the order of actions. If a person can understand the first pictogram, it is reasonable to expect he or she could understand the second one as well. Therefore, we selected a set of representative pictograms to minimize redundancy and maximize the inclusion of unique elements. To avoid discomfort, we excluded the pictograms illustrating private parts of human body. At the end, 20 pictograms remained in the test sample set. Figure 2 displays the pictogram images and their official textual interpretations.

### Designing the Web-Based Survey

**Figure 3:**
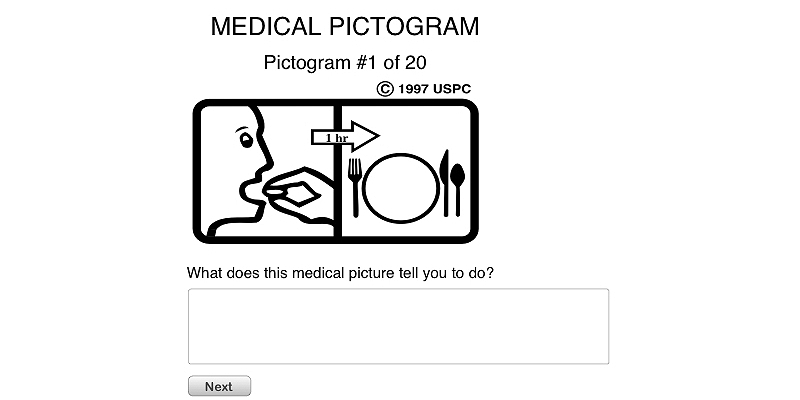
Interface of survey part I.

**Figure 4:**
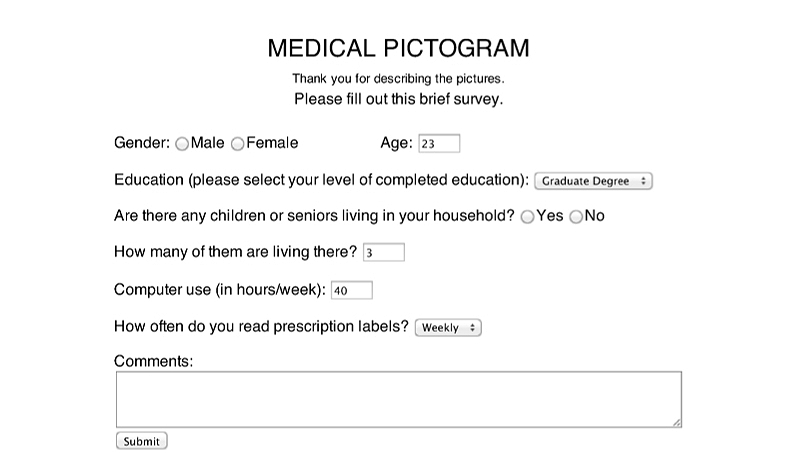
Interface of survey part II.

We designed and implemented a Web-based survey in the form of open-ended tests. We chose the free Web service provided by the Google App Engine to develop and host the survey app. The survey included 20 guesses, one for each test pictogram, followed by a short demographic questionnaire at the end. For each guess, the turkers viewed a medical pictogram and answered a question: “What does this medical picture tell you to do?” by typing in their responses in the textbox (see Figure 3). The demographic questionnaire asked for the participants’ gender, age, educational level, number of children or senior members in household, frequency of computer use, and frequency of reading medical labels (see Figure 4).

In addition to the textual responses, we recorded the turkers’ IP addresses for the purpose of removing redundant responses because some turkers may have created multiple accounts to be able to perform the same task multiple times in order to earn more. Of course, more than one turker may share a computer, resulting in identical IP addresses in multiple records. However, we should be able to distinguish these turkers by checking the differences in their pictogram interpretations and their answers to the demographic questions.

Once a participant finishes the entire survey, the survey app generates a random eight-digit code. The participants should submit this code to MTurk upon completion to verify that they went through the whole survey procedure and to receive payment.

### Setting Up the Task on MTurk

We created an MTurk requestor account to deploy the task, titled “Guess what the image tells you.” Turkers would also see a brief description: “Please view each image and write an instruction of what the image is telling you to do and answer the short survey at the end.” A turker was paid US$0.30 for interpreting the 20 pictograms and completing the short demographic questionnaire. The requested number of turkers for this task was set to 100.

To avoid spammers, we screened the turkers by setting the minimum prior approval rate to 95%. We also restricted the participants’ location to the United States. Considering that previous studies have shown that cultural backgrounds and ethnicity can have significant effects on people’s comprehension of pictograms [4,10], it is our future work to extend this survey to turkers outside the United States to study the effect of cultural backgrounds.

## Results

### Data Quality Control

We collected the required number of responses within 3 days. We received 104 responses in total, which means 4 respondents were not paid because they did not submit the confirmation code. We ran the following investigation to ensure the quality of the data. First, we checked for duplicate records. After sorting the data by participants’ IP addresses, we found three pairs of responses with the same IP address. In two pairs, the pictogram interpretations and the demographic survey answers were nearly identical, but the participation dates were different. We counted them as duplicate records and kept only the first record of each on file. In the third pair, the answers were different but valid; the second participation record was not paid. It is most likely that someone else “on-site” with that participating turker took the survey voluntarily but did not submit the code. In this case, we kept both records. Second, we identified the unpaid “volunteers”. We checked to see which random codes assigned by our app were not submitted to MTurk—these were unsolicited volunteers who might have happened to find our website through the participating turkers and who did the survey out of curiosity. We found 4 such “volunteers”, one of whom used the same IP address as 1 participating turker. An examination of the volunteers’ answers shows that they were not spammers; therefore, we kept their answers in the dataset. Finally, we manually checked the quality of all responses. Only 1 participant was identified as a spammer; this record was deleted from the dataset.

At the end of the process, the data that remained included responses from 101 valid participants. The data are publicly available for download (see Multimedia Appendix 1).

### Coding Open-Ended Interpretations

The comprehension test method we used in this study is open-ended testing, which is easier to construct and more accurate than multiple-choice testing, and is considered as a gold-standard in measuring symbol comprehension [19,20]. However, since the answers given by participants are usually short and ambiguous, it is difficult for judges to score them as either correct or incorrect [21]. To facilitate judges’ scoring the correctness of an interpretation and improving the reliability of their scoring, we used the 4-point rating scale (see Table 1). For example, for pictogram “take 1 hour before meals”, the interpretation “take the pill one hour before eating” would be rated as 1; “take before eating” as 2; “take medicine with food” as 3; and “take one hour after eating” as 4.

Two coders independently rated all the interpretations. The intercoder agreement was 0.83, based on the Krippendorff’s alpha measurement, demonstrating a high concordance between the 2 coders. A review of the disagreements showed that a large portion of the discrepancies were caused by the coders’ judgments on whether to make inferences about the implicit meaning in the responses. For example, for pictogram “take in the morning”, a number of participants gave interpretations like “take upon waking up”. In this case, one coder rated it 3 and the other, 2.

**Figure 5:**
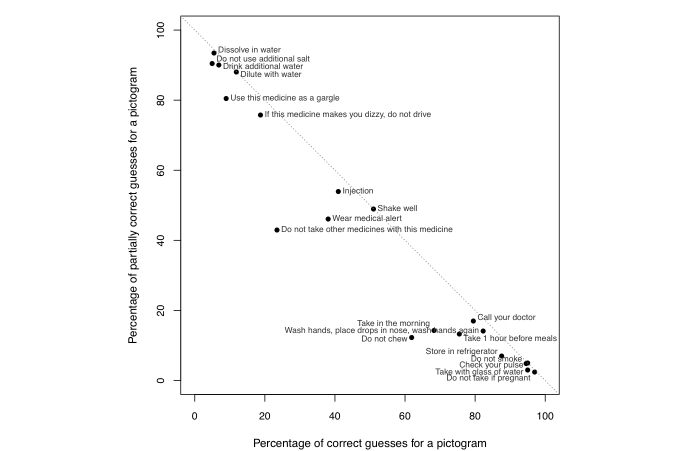
Distribution of correct and partially correct interpretations for the 20 pictograms.

For each pictogram, we calculated the percentages of correct, partially correct, and incorrect (wrong or completely wrong) guesses by each coder’s assessment and then averaged the percentages over the 2 coders (see Figure 2). On average, 52.4% of the interpretations were correct, 40.2% were partially correct, and 7.4% were incorrect. To help readers better understand the distribution of correct, partially correct, and incorrect guesses, Figure 5 presents a visualization of the distribution for each of the 20 pictograms. If a pictogram falls on the dotted diagonal line, it means all of its interpretations are either correct or partially correct; all pictograms under the diagonal line received at least one incorrect guess.

**Table 1:** Criteria for judging open-ended interpretations.

Rating	Category	Criteria
1	Correct	The interpretation is the same as, or very close to, the official description. The description maintains the important meaning and semantics of the official description.
2	Partially correct	The interpretation misses some information, or adds information not included in the official description. However, the discrepancies are minor.
3	Wrong	The interpretation is very different from the official description; it is difficult to understand or is confusing.
4	Completely wrong	The interpretation has no resemblance to the official description. It is completely wrong.

### Estimating Comprehensibility of Pictograms

Comprehensibility is usually estimated as the percentage of correct answers given by participants. However, as shown in Figures 2 and 5, 40% of guesses were partially correct in our data. To differentiate correct and partially correct guesses, we adopted ISO’s symbol testing procedures, which count partially correct guesses as a fraction in the total correct [20]. For the sake of simplicity, we counted each partially correct answer as 0.5 correct. With this treatment, the comprehensibility scores for the 20 pictograms ranged from 45% to 98%, with an average of 72.5% (Figure 2).

There have been several studies on the comprehensibility of the US pharmacopeial pictograms conducted with local people in South Africa [4], Finland [22], Portugal [23], and Hong Kong [24]. Among the four studies, the Portuguese one used multiple-choice test method—a method that could lead to an inflation of 30% in the comprehension scores when distractor alternatives were less plausible [25]. For the other three studies that used open-ended testing, 10 pictograms in the Hong Kong study, 7 pictograms in the Finland study, and 5 pictograms in the South Africa study, were the same or very similar to the ones that we used. Thus, we can conduct a comparison with the Hong Kong study (the study with the closest pictograms to ours). The education background of the participants in the Hong Kong study is also the closest to ours: 81% postsecondary education in the Hong Kong study and 92% in our study. In contrast, the participants in the Finland study were children, and the participants in the South Africa study were low-literate. Note that the Hong Kong study used a different scoring mechanism: 3 judges marked each response as either correct or incorrect, and the final decision would be correct or incorrect in case of perfect agreement and 0.5 correct otherwise.

**Figure 6:**
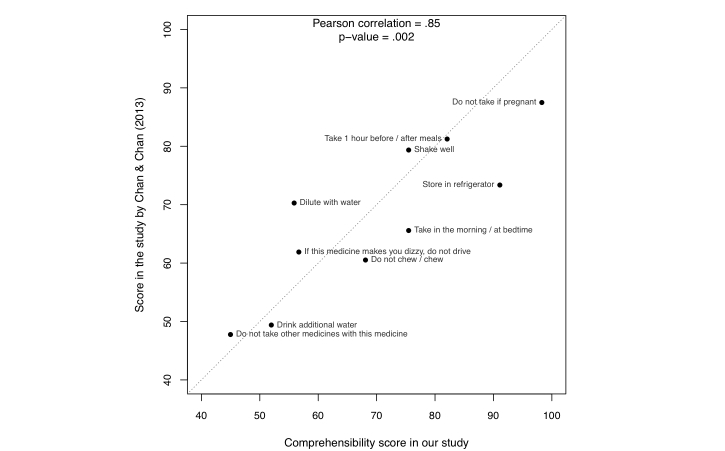
Comparison on 10 pictograms between our study and the Hong Kong study.

Figure 6 shows the comparison between our study and the Hong Kong study on 10 pictograms. In the figure, the dotted diagonal line represents that any pictogram falling on the line receives the same score from the two studies. The Pearson correlation between the two studies was .85 (*P*=.002). The strong correlation suggests that the pictogram evaluation result, which was obtained through recruiting online turkers to type in responses, is comparable to the result from recruiting local people to provide oral responses to open-ended tests.

### Content Analysis of Common Misinterpretations

**Figure 7:**
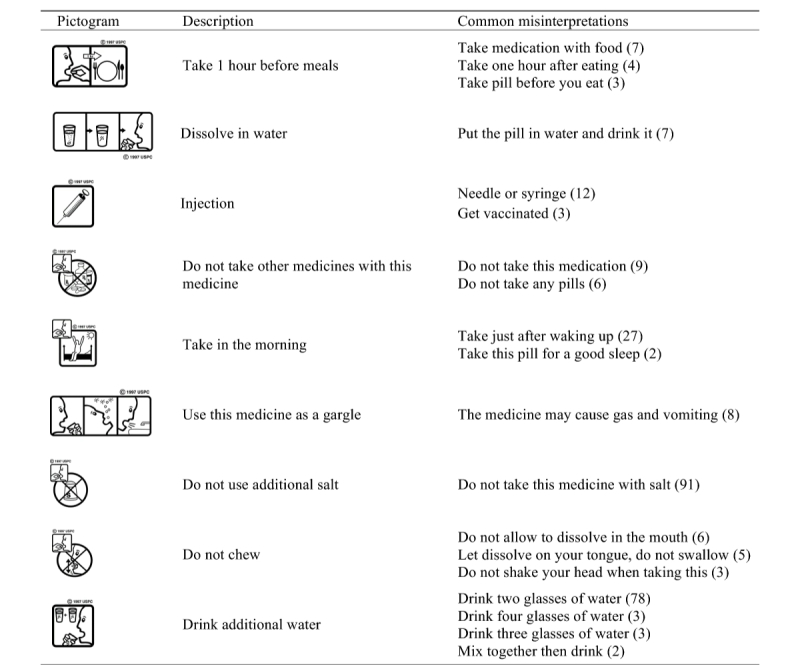
Common misinterpretations.

Figure 7 lists common misinterpretations (similar misinterpretations given by at least 2 turkers) for 9 pictograms. The number in the parentheses shows how many turkers described the pictogram in a similar way. For instance, in pictogram “take 1 hour before meals”, 7 participants described it as “take with food”, and 4 interpreted as “take 1 hour after food”. Such critical misinterpretations may well explain why Mansoor and Dowse added clocks in their redesign of the pictogram to prevent people from making the time order error [4,11].

An observation from these common misinterpretations is that some concepts are difficult to represent graphically. For example, it is hard to represent the modifier “additional” in the text “do not use additional water” or “drink additional water”, the verb “chew” in the text “do not chew”, and “morning” in “take in the morning”. To solve this problem, pictogram designers would use alternative strategies such as semantic associations [10,26]. In pictogram “drink additional water”, the US Pharmacopeial Convention used two (extra) glasses of water as an example to represent “additional water”. However, the concept “additional water” was still misunderstood as literally two glasses, three glasses (including the one in hand), or even four glasses (illusion). Hence, semantic associations may not be reliable because their interpretation depends on whether the underlying association or analogy can be identified by users.

Overall, the content analysis result showed that turkers’ misinterpretations shared common errors that exposed design problems in the tested pictograms. This finding validates the utility of the crowdsourcing approach for the participatory evaluation of medical pictograms.

### Participant Demographics and Pictogram Comprehension Score

Table 2 shows the demographic characteristics of the turkers in our study and the relationships between the turkers’ demographics and their comprehension performance. The gender distribution in our study, in which females accounted for 63%, was similar to the general US turker population that Ipeirotis reported in 2010 [27], in which females accounted for 65%. The age distribution was slightly different, with turkers aged 35 years or more accounting for 51% in our study and 45% in their study. The education distribution was also slightly different, with turkers who had college degrees or above accounting for 59% in our study and 54% in their study.

We used a two-tailed *t* test to compare male and female performance, and Spearman rank correlation to measure the correlations between the other ordinal demographic factors and participant performance. Females performed slightly better than males, but the difference was not significant (*P*=.078). Turkers with higher levels of education had better comprehension scores (Spearman rho=.25, *P*=.013), which is consistent with previous studies [28,29]. Educational level is the only factor that affected participant performance.

We also used Gamma test to measure the correlations among the ordinal demographic factors. No correlation was found except an interesting but not surprising one, which was that the number of children or seniors living in a household was negatively correlated with the frequency of computer use (gamma=-.33, *P*=.001).

**Table 2:** Participant demographics and pictogram comprehension score.

		Proportion	Comprehension
**Gender (n=97)**			*P*=.078
	Male	.37	70.7
	Female	.63	73.1
**Educational level (n=99)**			*P*=.013
	High school	.08	69.2
	Some college	.32	71.4
	College degree	.37	72.3
	Above college degree	.22	75.0
**Age (n=97)**			*P*=.54
	≤25	.13	69.5
	26-35	.36	73.4
	36-45	.14	69.9
	46-55	.21	74.5
	≥56	.16	72.1
**Hours of computer use per week (n=99)**			*P*=.69
	≤10	.11	71.1
	11-20	.25	72.2
	21-30	.16	73.8
	31-40	.19	72.4
	≥41	.28	72.1
**Prescription reading frequency (n=99)**			*P*=.81
	Daily	.15	73.0
	Weekly	.19	71.5
	Monthly	.23	74.1
	Every several months	.25	70.9
	Never	.17	72.7
**Number of children and seniors (n=83)**			*P*=.70
	0	.41	71.8
	1	.24	73.2
	2	.22	71.0
	≥3	.13	73.4

## Discussion

### Principal Findings

Our study aimed to assess whether MTurk, a popular crowdsourcing platform, can be used for participatory evaluation of medical pictograms. We recruited 100 US turkers to guess the meaning of 20 US Pharmacopeial Convention pictograms. The comprehensibility score for the 20 tested pictograms ranged from 45% to 98%, with an average of 72.5%. The scores of 10 pictograms were strongly correlated to the scores of the same pictograms reported in another study that used oral response-based open-ended testing with local people [24]. The turkers’ misinterpretations shared common errors that exposed design problems in the tested pictograms. These results demonstrate that MTurk can be an effective and inexpensive tool for evaluating pictograms and identifying problems in the design of medical pictograms.

We also investigated whether demographic factors (gender, age, educational level, etc) affect participant performance. We found that turkers with higher levels of education had better comprehension performance—a result consistent with previous studies on the effect of education [28,29].

### Limitations

A limitation of our study, which was also discussed by Turner et al regarding the use of MTurk in health communication [14], is that turkers, with relatively higher levels of education, may not be a representative sample of the general population. However, large crowds like MTurk are certainly more representative and cost-effective than the convenience samples in traditional participatory studies, which may consist of as few as 10 to 20 participants due to time and resource constraints [10,12]. To better understand the crowd’s representativeness, further investigation is needed to examine to what extent turkers’ misunderstandings overlap with those elicited from low-literate people. In addition, the problem of lack of less-educated participants may be greatly reduced when MTurk is available on smart phones, since ethnic minorities and less-educated people, according to the Pew Internet and American Life Project, primarily use their phone for Web access [30].

Another limitation is that our current study recruited only US turkers, and thus we could not conduct any analysis of the effects of cultural backgrounds, an important factor in pictogram comprehension [4,10]. One advantage of recruiting participants from MTurk is that one can recruit turkers from different countries with a variety of languages and cultural backgrounds [14]. It will be our future work to use this advantage to study the effects of cultural backgrounds by recruiting turkers from different countries.

### Future Work

We envision building a crowdsourcing tool that allows a large number of Internet users to design and evaluate medical pictograms. In this paper, we focused only on crowdsourced evaluation; in the future, we plan to recruit online users to participate in the design of medical pictograms. Existing work on crowd design, which asked turkers to iteratively sketch, evaluate, and combine the designs of chairs for children, has shown that a crowd-based design process can also be effective [31]. It will be interesting to study how the crowd can be effectively organized and motivated to design high-quality medical pictograms and how the crowdsourcing approach could complement automated illustration of patient instructions [32].

Another interesting direction is to investigate the potential of asking the crowd to evaluate volumes of open-ended interpretations. Open-ended testing is the method recommended by ANSI [19], but it is time-consuming and tedious for judges to score a large number of interpretations—in our case each judge needed to assess about 2000 interpretations. Clearly, turkers can also be recruited for evaluating the interpretations entered by their peers. Existing studies have shown that the crowd can perform well on various annotation tasks, and actually they may perform even better than experts as a result of collective wisdom [18,33,34].

## Multimedia Appendix 1

Collected survey data.

## References

[1] U.S. Department of Health and Human Services (2010). Healthy People.

[2] Katz MG, Kripalani S, Weiss BD (2006). Use of pictorial aids in medication instructions: a review of the literature. Am J Health Syst Pharm.

[3] Choi J (2011). Literature review: using pictographs in discharge instructions for older adults with low-literacy skills. J Clin Nurs.

[4] Dowse R, Ehlers MS (2001). The evaluation of pharmaceutical pictograms in a low-literate South African population. Patient Educ Couns.

[5] Dowse R, Ehlers M (2005). Medicine labels incorporating pictograms: do they influence understanding and adherence?. Patient Educ Couns.

[6] Houts PS, Doak CC, Doak LG, Loscalzo MJ (2006). The role of pictures in improving health communication: a review of research on attention, comprehension, recall, and adherence. Patient Educ Couns.

[7] Morrow DG, Leirer VO, Andrassy JM (1996). Using icons to convey medication schedule information. Appl Ergon.

[8] Ngoh LN, Shepherd MD (1997). Design, development, and evaluation of visual aids for communicating prescription drug instructions to nonliterate patients in rural Cameroon. Patient Educ Couns.

[9] Zeng-Treitler Q, Kim H, Hunter M (2008). Improving patient comprehension and recall of discharge instructions by supplementing free texts with pictographs. AMIA Annual Symposium Proceedings.

[10] Kim H, Nakamura C, Zeng-Treitler Q (2009). Assessment of pictographs developed through a participatory design process using an online survey tool. J Med Internet Res.

[11] Mansoor LE, Dowse R (2004). Design and evaluation of a new pharmaceutical pictogram sequence to convey medicine usage. Ergonomics SA.

[12] Ruland CM, Starren J, Vatne TM (2008). Participatory design with children in the development of a support system for patient-centered care in pediatric oncology. J Biomed Inform.

[13] Swan M (2012). Crowdsourced health research studies: an important emerging complement to clinical trials in the public health research ecosystem. J Med Internet Res.

[14] Turner AM, Kirchhoff K, Capurro D (2012). Using crowdsourcing technology for testing multilingual public health promotion materials. J Med Internet Res.

[15] Luengo-Oroz MA, Arranz A, Frean J (2012). Crowdsourcing malaria parasite quantification: an online game for analyzing images of infected thick blood smears. J Med Internet Res.

[16] Heer J, Bostock M (2010). Crowdsourcing Graphical Perception: Using Mechanical Turk to Assess Visualization Design. Proceedings of the 28th Annual ACM SIGCHI Conference on Human Factors in Computing Systems.

[17] Paolacci G, Chandler J, Ipeirotis PG (2010). Running experiments on Amazon Mechanical Turk. Judgment and Decision Making.

[18] Buhrmester M, Kwang T, Gosling SD (2011). Amazon's Mechanical Turk: A New Source of Inexpensive, Yet High-Quality, Data?. Perspectives on Psychological Science.

[19] American National Standards Institute (2007). American National Standard Criteria for Safety Symbols. ANSI Z535.3.

[20] Wogalter M, Silver N, Leonard S, Zaikina H, Wogalter M (2006). Warning symbols. Handbook of warnings.

[21] Lesch MF, McDevitt JR (2002). Methodological Issues in Testing Comprehension of Safety Symbols. Proceedings of the 46th Annual Human Factors and Ergonomics Society Annual Meeting.

[22] Hämeen-Anttila K, Kemppainen K, Enlund H, Bush Patricia J, Marja A (2004). Do pictograms improve children's understanding of medicine leaflet information?. Patient Educ Couns.

[23] Soares MA (2013). Legibility of USP pictograms by clients of community pharmacies in Portugal. Int J Clin Pharm.

[24] Chan AH, Chan KW (2013). Effects of prospective-user factors and sign design features on guessability of pharmaceutical pictograms. Patient Educ Couns.

[25] Wolff JS, Wogalter MS (1998). Comprehension of Pictorial Symbols: Effects of Context and Test Method. hum factors.

[26] Nakamura C, Zeng-Treitler Q (2012). A Taxonomy of Representation Strategies in Iconic Communication. Int J Hum Comput Stud.

[27] Ipeirotis P (2010). Demographics of Mechanical Turk. New York University Tech Report.

[28] Dowse R, Ehlers MS (2003). The influence of education on the interpretation of pharmaceutical pictograms for communicating medicine instructions. International Journal of Pharmacy Practice.

[29] Richler M, Vaillancourt R, Celetti SJ, Besançon L, Arun K, Sebastien F (2012). The use of pictograms to convey health information regarding side effects and/or indications of medications. Journal of Communication In Healthcare.

[30] Fox S, Duggan M (2012). Pew Research Center.

[31] Yu L, Nickerson JV (2011). Cooks or Cobblers? Crowd Creativity through Combination. Proceedings of the 29th Annual ACM SIGCHI Conference on Human Factors in Computing Systems.

[32] Bui D, Nakamura C, Bray BE, Zeng-Treitler Q (2012). Automated illustration of patients instructions. AMIA Annual Symposium Proceedings.

[33] Snow R, O’Connor B, Jurafsky D, Ng AY (2008). Cheap and fast--but is it good?: evaluating non-expert annotations for natural language tasks. Proceedings of the Conference on Empirical Methods in Natural Language Processing.

[34] Galton F (1907). Vox Populi. Nature.

